# Desert Hedgehog/Patch2 Axis Contributes to Vascular Permeability and Angiogenesis in Glioblastoma

**DOI:** 10.3389/fphar.2015.00281

**Published:** 2015-11-24

**Authors:** Sandy Azzi, Lucas Treps, Héloïse M. Leclair, Hai-Mi Ngo, Elizabeth Harford-Wright, Julie Gavard

**Affiliations:** ^1^INSERM, U1016, CNRS, UMR8104, Université Paris Descartes, Paris, France; ^2^INSERM, U892, CNRS, UMR6299, Université de Nantes, Nantes, France

**Keywords:** glioma, brain endothelial cells, hedgehog, permeability, tumor vasculature

## Abstract

Glioblastoma multiforme (GBM) constitutes the most common and the most aggressive type of human tumors affecting the central nervous system. Prognosis remains dark due to the inefficiency of current treatments and the rapid relapse. Paralleling other human tumors, GBM contains a fraction of tumor initiating cells with the capacity to self-renew, initiate and maintain the tumor mass. These cells were found in close proximity to brain vasculature, suggesting functional interactions between brain tumor-initiating cells (BTICs) and endothelial cells within the so-called vascular niche. However, the mechanisms by which these cells impact on the endothelium plasticity and function remain unclear. Using culture of BTICs isolated from a cohort of 14 GBM patients, we show that BTICs secretome promotes brain endothelial cell remodeling in a VEGF-independent manner. Gene array analysis unmasked that BTICs-released factors drove the expression of Ptch2 in endothelial cells. Interestingly, BTICs produce desert hedgehog (DHH) ligand, enabling a paracrine DHH/Ptch2 signaling cascade that conveys elevated permeability and angiogenesis. Finally, DHH silencing in BTICs dramatically reduced tumor growth, as well as vascularization and intra-tumor permeability. Collectively, our data unveil a role for DHH in exacerbated tumor angiogenesis and permeability, which may ultimately favor glioblastoma growth, and thus place the DHH/Ptch2 nexus as a molecular target for novel therapies.

## Introduction

Glioblastoma multiforme (GBM) is one of the most malignant cancers in adults with less than 5% of patients surviving more than 2 years and a median survival range between 15 and 18 months (Stupp et al., 2009). While current therapies include surgical resection, followed by a combined radio-chemotherapy regime (Stupp et al., 2009), relapse is almost inevitable and remains fatal. GBM are highly vascularized tumors, with large zone of hypoxia and necrosis and are highly addicted to pro-angiogenic signaling pathways, among which is the vascular endothelial growth factor A (VEGF-A; Olsson et al., 2006; Jain et al., 2007). Vascularization index, blood flux, and vascular permeability and edema are thus important parameters directly linked to tumor aggressiveness (Jain et al., 2007; Sorensen et al., 2009). Echoing this, the humanized anti-VEGF antibodies bevacizumab, as well as novel anti-angiogenic therapies were developed to prune and/or normalize the tumor blood supply (Carmeliet and Jain, 2011). However, recent randomized large clinical studies failed to demonstrate the effectiveness of such therapies in first line treatment in GBM (Chinot et al., 2014). In this context, identification of new molecular targets controlling endothelial plasticity is at the utmost importance, as it may open new routes for more efficient therapeutic strategies in GBM treatment.

The identification of cancer cells with stem-like properties has contributed to a better knowledge of molecular and cellular processes involved in tumor formation and treatment resistance (Singh et al., 2003, 2004; Bao et al., 2006). Indeed, GBM are believed to originate from such brain tumor-initiating cells (BTICs) with the unique ability to self-sustain and renew the tumor (Singh et al., 2003, 2004; Bao et al., 2006). Such studies have highlighted the importance of both cellular hierarchy and intratumoral heterogeneity in GBM (Verhaak et al., 2010; Brennan et al., 2013; Patel et al., 2014). Similarly to neural stem cells, BTICs are nested in a vascular niche, which provides a specific and confined microenvironment that favors bidirectional transference between endothelial cells from the vascular wall and cancer initiating cells from the tumor mass. In keeping with this, BTICs were characterized in the vicinity of brain endothelial cells, allowing close interaction and exchange of survival and fate signals (Calabrese et al., 2007; Galan-Moya et al., 2011). Several studies had also documented the ability of BTICs to transdifferentiate and integrate the tumor vasculature (Ricci-Vitiani et al., 2010; Wang et al., 2010; Cheng et al., 2013). However, the precise mechanism by which BTICs impact on endothelial plasticity remains poorly understood.

Among the putative candidates, the hedgehog (HH) pathway is known to regulate stem cell maintenance, differentiation and proliferation in embryonic development. Notably, the HH network coordinates the blood–brain barrier integrity in both development and adulthood in mouse models (Alvarez et al., 2011). In pathological conditions, HH aberrant activation was linked to tumor invasion, migration, and progression (Smyth et al., 1999; Evangelista et al., 2006; D’Amico et al., 2015; Qualtrough et al., 2015; Xu et al., 2015). Moreover, HH signaling was found implicated in acquired chemoresistance and cancer stem cell fate and properties (Bar et al., 2007; Justilien et al., 2014; Kong et al., 2014; Takebe et al., 2015).

In the present work, we investigated the effects of patient-derived BTICs-secreted factors on long-term endothelial cell behavior. We found that BTICs secretome conveys endothelial remodeling in a VEGF/Src-independent manner. We discovered that BTICs-secreted factors induce the expression of the HH receptor Ptch2, in endothelial cells. Interestingly, BTICs produce desert hedgehog (DHH) ligand, enabling a paracrine DHH/Ptch2 signaling cascade that culminates in vascular leakage and increased angiogenic potential. Finally, DHH knockdown in BTICs dramatically reduced tumor formation and growth, as well as intra-tumor permeability. Thus, our data place DHH/Ptch2 nexus as a potential candidate for therapeutic intervention in GBM.

## Materials and Methods

### Cell Culture

Immortalized human cerebral microvascular endothelial cells (hCMEC/D3) were maintained as described previously in (Le Guelte et al., 2012). Immortalized human umbilical vein endothelial cells (HUVEC, Ea.hy926 clone) and human embryonic kidney HEK-293T cells were obtained from ATCC (LGC Standards, Molsheim, France) and expanded in DMEM supplemented with 10% fetal bovine serum (FBS) and 1% penicillin/streptomycin (Life Technologies, Cergy-Pontoise, France). Human bone marrow endothelial cells (hBMEC) were obtained from (Lonza, Levallois, France) and maintained in complete EBM-2 medium, as per manufacturer’s instructions (Lonza).

Patient-derived glioblastoma-initiating cells (BTICs) were isolated from primary glioblastoma tumor biopsies and cultured as tumorspheres as previously described (Galan-Moya et al., 2011; Treps et al., 2015). BTICs-derived conditioned media were prepared from 1.10^6^ cells cultured in DMEM/F12 additive-free medium. Supernatants were collected 3 days later, filtered on a 0.45 μm membrane and stored at –80°C.

### Animals

This study was carried out in accordance with the recommendations of local ethics committee (Paris Descartes university, Paris, France) and approved by French Ministry of Research (agreement number #00754.02). All experiments were performed in compliance with the European Convention for the Protection of Vertebrate Animals used for Experimental and other Scientific Purposes (ETS 123). Six weeks-old female BALB/c nude mice were obtained from Janvier Labs (Saint-Berthevin, France).

### Reagents and Secondary Antibodies

Recombinant VEGF-A_165_ was purchased from R&D Systems (Bio-Techne, Lille, France). VEGF-R2 inhibitor (SU5416) and Src kinase inhibitor (SU6656) were from Tocris (Bio-Techne). Hypoxyprobe Red549 Kit was obtained from Hypoxyprobe (Burlington, MA, USA). Growth factor-free matrigel was from BD Biosciences (Le Pont-de-Claix, France). FITC-coupled 40 kDa Dextran and Alexa488-coupled phalloidin were from Life Technologies. HRP-conjugated and fluorochrome-conjugated specie-specific secondary antibodies were purchased from Jackson Immunoresearch (Suffolk, UK) and Life Technologies, respectively.

### siRNA Transfection and shRNA Retroviral Transduction

For transient gene silencing, 25 nM of siRNA were transfected using Lipofectamine RNAiMax (Life Technologies). The following silencing duplexes were used: predesigned MISSION siRNA against human DHH (CUUGCUACGCGGUUCUGGA; Sigma, St-Quentin-Fallavier, France), duplexes against human PTCH1 (HSS108758, Life Technologies) and human PTCH2 (HSS112667, Life Technologies), and non-silencing control siRNA (sic, Life Technologies). Preparation of sic and DHH siRNA-transfected BTICs-conditioned media (BTICs-CM) was performed 2 days post-transfection.

Stable knockdown of DHH was performed in retrovirally-infected BTICs as described previously (Dubois et al., 2014). Briefly, HEK-293T cells were transfected with a mixture of pVSVg, pSPAX2, and pGIPZ-GFP or pGIPZ-GFP-DHH shRNA (Thermo Fisher Scientific, Illkirch, France). Supernatants were collected 2 days post-transfection, clarified by centrifugation and used to transduce BTICs. Transduction and knockdown efficiency were checked by flow cytometry and RT-PCR, respectively, prior implantation in animals.

### Gene Array Analysis

Monolayers of quiescent hCMEC/D3 cells were exposed for 24 h to four different patient-derived BTICs-derived condition media. Endothelial RNAs were purified (Qiagen, Courtaboeuf, France) and labeled (Ambion) for transcriptome (Affymetrix, eBioscience, Hatfield, UK) and statistical analysis (Genom’IC core facility, Institut Cochin, Paris, France).

### Reverse Transcription-Polymerase Chain Reaction

Purified RNA (Qiagen) were processed for reverse transcription using the Maxima RT Kit (Thermo Fisher) and used to amplify human PTCH2, PTCH1, DHH, DHRS3, ODZ2, EBF4, LPPR5, RHOU, NXPH4, GAPDH, and BACT. RedTaq Ready Mix (Sigma) and specific primer sets were used for PCR, as described in (Leclair et al., 2014).

### Flow Cytometry Analysis

Ptch1 and Ptch2 expression were analyzed using an indirect immunofluorescence protocol. Cells were incubated with primary antibodies (Ptch2 SAB2101905 and Ptch1 AV44249, Sigma), washed three times with cold phosphate-buffered saline and further stained with the corresponding Alexa488-conjugated IgG (Life Technologies). Data were acquired on a FACScalibur (CellQuest software; BD) and analyzed using FlowJo (Ashland, OR, USA).

### ELISA

Desert hedgehog (DHH) and Sonic hedgehog (SHH) secreted concentrations were analyzed by ELISA assay as described in (Azzi et al., 2010). Briefly, 50 μl of BTICs-CM were coated in a 96 well ELISA plate at 4°C overnight, and saturated (BSA 3%, 30 min). Primary antibodies (DHH SAB1407419 and SHH SAB2108581, Sigma), were then added, washed five times with a PBS-Tween 0.5% solution, and further stained with the corresponding HRP-conjugated IgG. Plates were washed five times, and 50 μl of TMB substrate (Sigma) was added for 20 min in the dark. Absorbance was read at 650 nm. Recombinant DHH (Emelca Bioscience) and SHH (Sigma) were used to establish the standard curves.

### Tubulogenesis Assay

Tubulogenesis assay was performed as previously described (Dwyer et al., 2012). Briefly matrigel was added to a 96-well plate and allowed to polymerize for 30 min at 37°C. hCMEC/D3 endothelial cells (10,000 cells/well) were pre-treated with BTICs-CM for 24 h, prior being seeded on top of matrigel. Images of at least five fields of view (FOV) per condition, randomly chosen, were acquired each hour over an 8-h period (Motic, AE21 microscope, Wetzlar, Germany). For tube formation and branches quantifications, images were processed using ImageJ software (NIH, Bethesda, MD, USA).

### Sprouting Assay

Sprouting of hCMEC/D3 cells was assessed as described in (Wimmer et al., 2012). Briefly, 4000 Cytodex3 microcarrier beads (Sigma) were coated with collagen (BD Biosciences), and mixed with 10^6^ hCMEC/D3 cells in warm EBM-2 medium. The mixture was incubated for 4 h at 37°C, with regular shaking (each 15 min). Coated beads were transferred to a new culture dish overnight to remove unattached cells, then washed with PBS and re-suspended in a 2.5 mg/ml fibrinogen-aprotinin (0.15 Units/ml, Sigma) solution + bFGF2 (200 ng/ml, Sigma). The mixture was then distributed in eight wells Ibidi plate containing 0.625 U/mL of thrombin, and allowed to clot for 15 min at 37°C. EBM-2 medium or BTICs-CM were added on top of the fibrin matrix and sprouting allowed for 3 days.

For imaging, matrix containing-beads were fixed (PFA 4%, 30 min), permeabilized (Triton 0.5%, 10 min), and saturated (BSA 3%, overnight at 4°C). Phalloidin and DAPI labeling were performed and images acquired using Leica fluorescence microscope (Imagery facility, Institut Cochin, Paris). Number of sprouting cells and sprout length, in at least five FOV randomly chosen, were quantified using ImageJ software.

### Permeability Assays

*In vitro* and *in vivo* permeability assays were conducted as described in (Gavard and Gutkind, 2006; Le Guelte et al., 2012; Treps et al., 2015). Briefly, for *in vitro* assay, 1.10^5^ hCMEC/D3 cells were seeded on 3 μm pore-size collagen-coated PTFE membranes (Costar, VWR, Fontenay-sous-Bois, France) for 3 days. Cells were then treated with BTICs-CM for 24 h, and permeability evaluated by FITC-dextran 40 kDa passage. Fluorescence was measured using the Fusion plate reader (Packard, San Diego, CA, USA). Data were normalized to untreated samples and expressed as the mean on three independent experiments. For *in vivo* miles assay, sterile Evans blue (1% in PBS, Sigma) was administrated by retro-orbital injection in ketamine/xylazine mixture (50 and 5 mg/kg) anesthetized animals. Mice were sacrificed 30 min later, and intra-tumor blue extravasation evaluated by absorbance. Results were normalized to skin punctures.

### Xenografts and Tissue Staining

Brain tumor-initiating cells transduced with shRNA against DHH or non-silencing shRNA control were re-suspended in a mixture of PBS/matrigel (1:1), and injected subcutaneously (1.10^6^ cells/injection) in the flank of BALB/c nude mice. Tumor formation and volume were assessed over the duration of the experiment and quantified using following the equation: volume = (width^2^ × length)/2 formula, in a double-blind study. To evaluate intra-tumor hypoxic zones, pimonidazole was administrated at 30 mg/kg by intravenous injection, 1 h prior mice sacrifice and tumor extraction. Tumors were cryopreserved in OCT to be later processed for imaging analysis.

Tissue sections were obtained using Leica cryostat (Histology core-facility, Cochin Institute, Paris, France). Sections were fixed (PFA 4% for 30 min), permeabilized (Triton 0.5%, 10 min), saturated (BSA 3%, 2 h) and incubated with CD31 antibody (1/200, overnight at 4°C, BD Biosciences). Tissue samples were washed and further incubated with corresponding FITC-coupled secondary antibody for 1 h. Alternatively, sections were incubated with Hp-Dylight^™^ 549 conjugated antibody overnight at 4°C. Images of five different FOV of three different sections were acquired using Leica fluorescence microscope, and staining quantified using the ImageJ software.

All statistical analyses were performed on two or three independent experiments, using Prism software (GraphPad, La Jolla, CA, USA).

## Results

### Secretome of Patient-Derived Glioblastoma-Initiating Cells Impacts on Brain Endothelial Cell Plasticity

**FIGURE 1:**
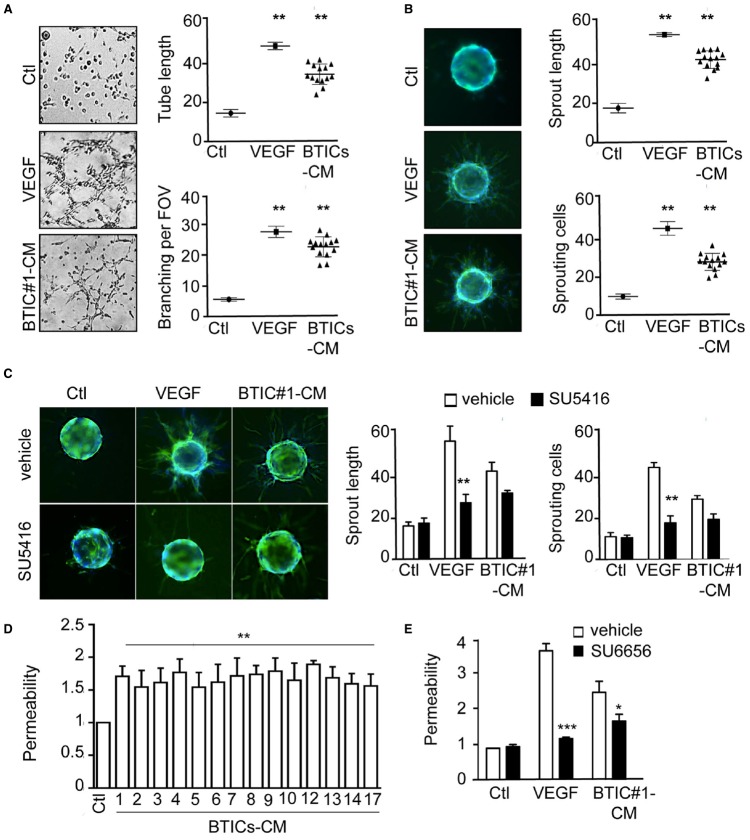
**Patient-derived BTICs-secreted factors induced brain endothelial cell remodeling, independently of the VEGF-R2/Src axis. (A)** Brain endothelial hCMEC/D3 cells were tested for tube formation capacity in response to VEGF and all 14 BTICs-derived conditioned media. Representative images are shown. Tube length and number of branch points per field of view (FOV) were quantified. **(B,C)** hCMEC/D3 cells sprouting ability was evaluated in the absence **(B)** or presence **(C)** of the VEGF R2 inhibitor SU5416 (5 μM). The number of sprouting cells and mean sprout length were determined. **(D,E)** Permeability of brain endothelial cells treated with BTICs-derived conditioned media. Cells were pre-treated with DMSO (Vehicle) or SU6656 (Src inhibitor, 1 μM) prior BTICs-CM treatments in **(E)**. Data are expressed as fold change relative to the control. ***p < 0.001, **p < 0.01, *p < 0.05 by analysis of variance (ANOVA). Each panel is representative of at least three independent experiments.

While previous studies had highlighted the importance of the vascular niche in maintaining the BTICs population (Calabrese et al., 2007; Galan-Moya et al., 2011), how in turn BTICs might impact on endothelial fate is poorly documented. We thus explore the effects of patient-derived BTICs-secreted factors on endothelial cell remodeling. To this aim, confluent monolayers of human endothelial brain endothelial cells (hCMEC/D3) cells were exposed to BTICs-CM for 24 h. BTICs-CM induced tubule formation and branching to the same extent as VEGF-A. Interestingly, this effect was significantly higher than serum-free EBM-2 medium (Ctl) and was recapitulated in all 14 patient-derived BTICs tested (Figure 1A). Accordingly, BTICs-CM promoted hCMEC/D3 sprouting from collagen-coated microcarriers (Figure 1B). Indeed, both sprout length and sprout number were elevated, when compared to control conditions, albeit slightly lower than VEGF-A-treated conditions (Figure 1B). As VEGF-A is a key driver of endothelial plasticity (Folkman, 2006; Gavard and Gutkind, 2006), we next assessed whether it was required for BTICs-CM-triggered angiogenic phenotype. While inhibiting VEGF-R2 tyrosine kinase activity resulted in a dramatic reduction in both sprouting length and number of sprouting cells upon VEGF-A stimulation, no overt changes were observed when exposed to BTICs-CM (Figure 1C). Furthermore, BTICs-CM heightened endothelial permeability, as indicated by a two-fold increase of FITC-dextran passage (Figure 1D), an effect seen in all the 14 GBM patient-derived BTICs tested. Again, it is unlikely this effect is dependent on VEGF-A, as blocking Src kinase activity; the main downstream regulator of VEGF-A-induced permeability (Eliceiri et al., 1999; Gavard and Gutkind, 2006); only partially reduced the BTICs-CM-triggered endothelial permeability increase (Figure 1E). Thus, our data suggest that BTICs-released factors induce endothelial cell remodeling in a VEGF-independent manner.

### Ptch2 Receptor is Upregulated in BTIC-CM-Exposed Endothelial Cells

**FIGURE 2:**
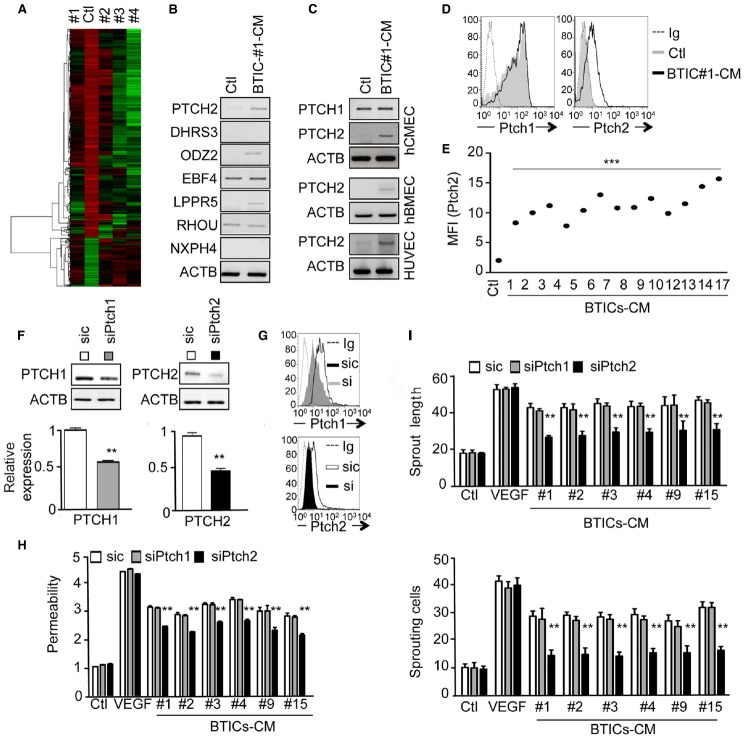
**The Hedgehog receptor Ptch2 is up-regulated in BTIC-CM-exposed brain endothelial cells. (A)** Gene array analysis of hCMEC/D3 cells exposed to BTIC-derived conditioned media for 24 h. Untreated endothelial cells were used as control. **(B)** Gene array confirmation by RT-PCR of seven up-regulated genes common to all treated conditions. Actin (ACTB) was used as a reference gene. **(C)** RT-PCR analysis of PTCH2 expression in three different types of endothelial cells. Confluent monolayers of hCMEC/D3, hBMEC, and HUVEC cells were exposed to BTIC-derived conditioned media for 24 h, and PTCH2 expression analyzed by RT-PCR. Actin (ACTB) was used as a reference gene. **(D,E)** hCMEC/D3 cells were analyzed by flow cytometry for total Ptch1 and Ptch2 expression before (Ctl) and after BTICs-derived conditioned media stimulation (BTIC-CM, 24 h). In **(D)**, dashed histograms correspond to the isotype-matched control (Ig) and gray and black histograms show antibody staining of control and stimulated conditions, respectively. Mean of fluorescence of 10,000 events (MFI) for Ptch2 expression in untreated (Ctl) versus stimulated (BTIC-CM) endothelial cells is shown in **(E)**. **(F–H)** hCMEC/D3 cells were transfected with control (sic) or siRNA against Ptch1 (siPtch1) or Ptch2 (siPtch2). For Ptch2 silencing, 48 h after receiving the siRNA, endothelial cells were further stimulated with BTIC-CM for another 24 h in order to induce Ptch2 expression. **(F)** Ptch1 and Ptch2 expression were analyzed by RT-PCR. Actin (ACTB) was used as a reference gene. **(G)** Ptch1 and Ptch2 expression were analyzed by flow cytometry. Dashed histograms correspond to the isotype-matched control (Ig) and gray and black histograms show antibody staining in endothelial cells treated as indicated. **(H)** Permeability of endothelial cells was assessed. **(I)** hCMEC/D3 cells were also tested for their sprouting capacity. The number of sprouting cells and mean sprout length were determined. ***p < 0.001, **p < 0.01 by analysis of variance (ANOVA). Each panel is representative of at least two independent experiments.

In order to gain further insights into the molecular mechanisms involved in BTICs-based action, a transcriptome analysis of endothelial cells exposed to four different BTICs-CM (#1, #2, #3, #4) was performed, and a heat map of up- and down-regulated genes was generated (Figure 2A). Statistical analysis of shared genes with modified expression of at least two fold, and a p value of <0.05 was performed revealing seven genes of interest. *PTCH2*, *DHRS3*, *ODZ2*, *EBF4*, *LPPR5*, *RHOU*, and *NXPH4* were found *in silico* to be significantly up-regulated in treated cells, when compared to untreated endothelial cells. An independent RNA analysis confirmed the up-regulation of: *PTCH2*, *ODZ2*, and *LPPR5* (Figure 2B). Among these three candidates, we next focused on *PTCH2*, one of the two HH receptors. Indeed, HH signaling is aberrantly activated in many cancers and contributes to both tumor (Smyth et al., 1999) and blood–brain barrier development (Alvarez et al., 2011). Moreover, HH signaling was reported to supervise tumor-initiating cell fate and phenotype (Bar et al., 2007; Justilien et al., 2014; Takebe et al., 2015). In this context, *PTCH2* expression was observed in three different endothelial cell lines, only upon BTICs-CM challenge (Figure 2C), suggesting that this effect was not restricted to brain microcirculation. Further flow cytometry analysis showed that at various levels, Ptch2 was upregulated in response to a large panel of BTICs-CM (Figures 2D,E), suggesting that Ptch2 up-regulation is not a BTICs-type dependent phenomenon. Interestingly, PTCH1 expression remained unchanged in control and treated cells (Figures 2C,D). To address the functional role of Ptch2 in endothelial cells, RNA interference was employed to prevent BTICs-CM-driven up-regulation (Figures 2F–G). When Ptch2 was no longer induced, endothelial permeability and sprouting were significantly impaired (Figures 2H,I). Interestingly, Ptch1 siRNA failed to phenocopy Ptch2 silencing (Figures 2H,I). Finally, endothelial responses to VEGF-A were left intact in terms of sprouting and permeability (Figures 2H,I), reinforcing the VEGF-independent property of BTICs-secreted factors.

### Desert Hedgehog Mediates Permeability and Angiogenesis in Brain Endothelial Cells

**FIGURE 3:**
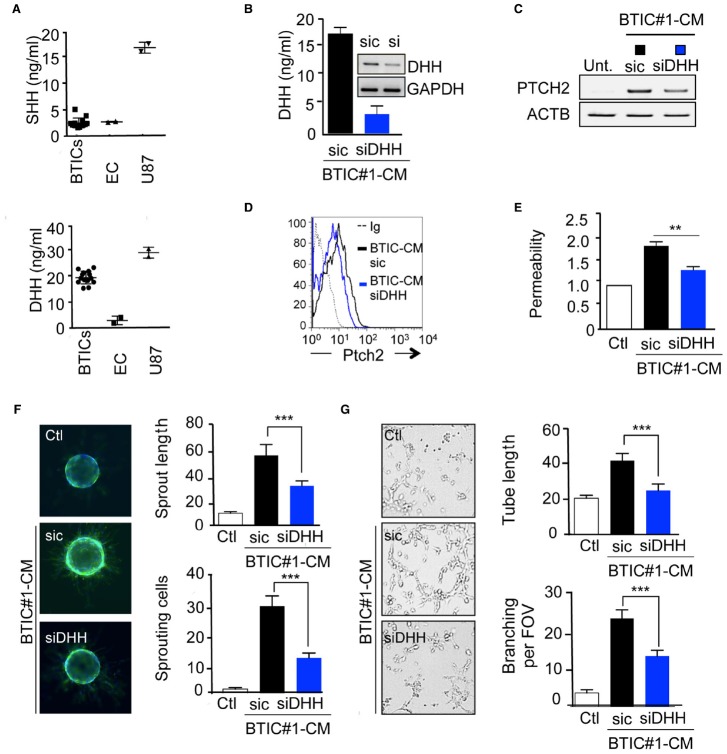
**DHH mediates the BTICs-CM effects in brain endothelial cells. (A)** SHH and DHH secretion was examined by ELISA in BTICs and endothelial cells (EC) conditioned media. U87 were used as a positive control. **(B)** BTICs cells were transfected with either control (sic) or siRNA against DHH (siDHH). Conditioned media was collected and processed for DHH secretion by ELISA assay. Alternatively, mRNA were extracted from both sic and siDHH receiving cells, and DHH expression was assessed by RT-PCR. GAPDH was used as a reference gene. **(C–G)** Confluent monolayers of hCMEC/D3 cells were exposed for 24 h to BTICs-conditioned media depleted (siDHH) or not (sic) for DHH. Ptch2 expression was assessed by RT-PCR **(C)** and flow cytometry analysis **(D)**. Dashed histograms correspond to the isotype-matched control (Ig), black and blue histograms show antibody staining in endothelial cells treated as indicated. **(E)** Permeability of endothelial cells was analyzed. **(F,G)** Endothelial cells were also tested for their sprouting **(F)** and tube formation **(G)** ability. The number of sprouting cells and mean sprout length were determined in **(F)**. Tube length, and number of branch points were counted in **(G)**. ***p < 0.001, **p < 0.01 by analysis of variance (ANOVA). Each panel is representative of at least two independent experiments.

We next investigated which of the three known ligands of Ptch2 could be released in BTICs-CM or in endothelial cell-CM. DHH was notably detected in the 14 different BTICs-CM at the average concentration of 15 ng/ml, while sonic hedgehog (SHH) could not be detected (Figure 3A). Because endothelial cells do not secrete any of these ligands, unlike U87 glioma cells, an autocrine action of HH ligands on brain endothelial cells was discarded (Figure 3A). Thus, we further investigated how BTICs-secreted DHH could modify endothelial homeostasis. Upon DHH silencing, BTICs-mediated Ptch2 upregulation was quelled, suggesting that Ptch2 could be its own direct target in a positive feedback loop, as demonstrated previously in HH signaling (Figures 3B,C,D; Evangelista et al., 2006; Aberger et al., 2012). Interestingly, BTICs-CM no longer induced permeability (Figure 3E), and depletion of DHH dramatically reduced the BTICs-CM pro-angiogenic effects, as assessed by sprouting and tubulogenesis ability (Figures 3F,G). Taken together, these results indicate that BTICs induce the expression of Ptch2 receptor in endothelial cells, while producing DHH ligand in the medium, thus enabling a paracrine DHH/Ptch2 signaling cascade that culminates in endothelial remodeling.

### Desert Hedgehog Drives Vascular Dysfunctions in Mice

**FIGURE 4:**
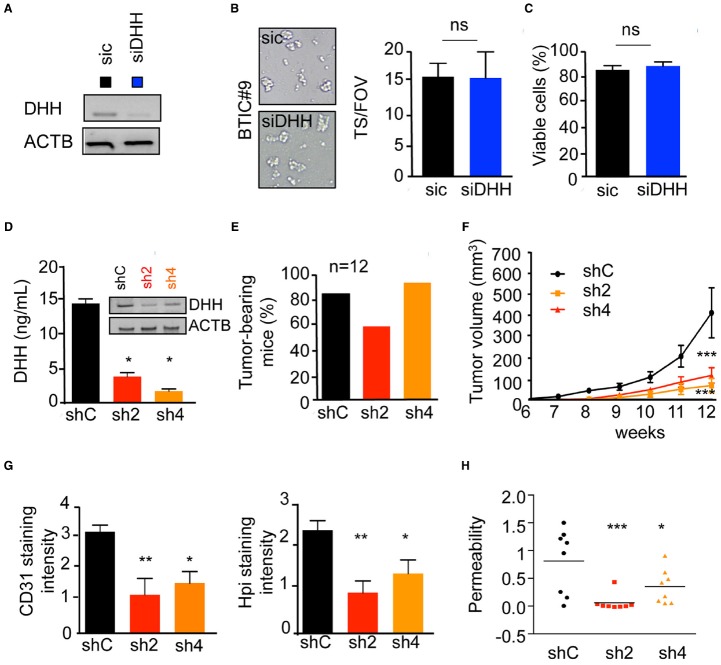
**DHH drives vascular dysfunctions ***in vivo***. (A–C)** BTIC#9 cells received either control (sic) or siRNA against DHH (siDHH). **(A)** Cells were processed for DHH expression by RT-PCR. ACTB was used as a reference gene. **(B)** BTICs cells were tested for their neurospheres formation capacity. Representative images are shown. The number of spheres per field of view (FOV) was quantified. **(C)** Cell viability was analyzed using trypan blue staining. **(D–H)** BTICs cells were stably transfected with control (shC) or two shRNAs against DHH (sh2 and sh4) plasmids. **(D)** DHH expression and secretion was analyzed by RT-PCR and ELISA. **(E)** BTICs cells were injected subcutaneously in each flank of nude mice, and tumor-baring mice counted 12 weeks later. **(F)** Tumor volume was evaluated. **(G)** Frozen tissue sections from obtained tumor xenografts were stained with CD31 and Hpi. Staining intensity was quantified using Image J. **(H)** Intra-tumor vascular permeability was measured using *in vivo* Miles assay. ***p < 0.001, **p < 0.01, *p < 0.05 by analysis of variance (ANOVA).

Finally, we explored the contribution of the paracrine DHH/Ptch2 signaling *in vivo*, using ectopic subcutaneous xenografts. Importantly, DHH silencing did not influence BTICs tumorspheres formation and viability (Figures 4A–C). Indeed, DHH-silenced BTICs maintained their self-renewal ability, as evidenced by the tumorspheres formation (Figure 4B), while their viability rate remained similar to control cells (Figure 4C). BTICs stably depleted for DHH expression were engineered and two clones (sh2 and sh4) were selected for their silencing efficiency and compared to cells that receive a non-silencing shRNA (shC). Accordingly, DHH expression and secretion were dramatically reduced in both sh2 and sh4 clones (Figure 4D). The reduction of DHH production had no significant effect on tumor initiation *in vivo*, as the number of tumor-bearing mice scored higher than 2/3 in each group within 12 weeks (Figure 4E). However, DHH loss-of-function in BTICs had a dramatic effect on tumor growth, as sh2 and sh4 clones produced tumors four times smaller than controls (Figure 4F). Likewise, vessels (CD31 staining) and hypoxic zones (Hpi staining) density were alleviated in DHH-less xenografts (Figure 4G). Accordingly, this effect was associated with a restrained intra-tumor vascular leakage (Figure 4H). Collectively, these results unveil a role for DHH in exacerbated tumor angiogenesis and permeability, which can ultimately favor glioblastoma growth, and thus place the DHH/Ptch2 nexus as a potential candidate for therapeutic intervention.

## Discussion

In summary, we report here that glioblastoma-initiating cells, which compose a small fraction of cells able to self-renew and drive tumorigenesis, promote brain endothelial cell remodeling though aberrant HH signaling. Indeed, we found that BTICs released DHH ligand in the extracellular space, while inducing Ptch2 expression in endothelial cells. Additionally, DHH ligand elicits a signaling cascade, through its receptor Ptch2, ultimately impacting on endothelial plasticity.

The HH family is composed of three members, Sonic (SHH), Desert (DHH), and Indian (IHH) that serve as ligands for PTCH receptors, Ptch1 and Ptch2 (Echelard et al., 1993; Riddle et al., 1993). Of note, the HH pathway is aberrantly activated in many cancers, and contributes both to tumor progression in various organs such as brain, lung, and skin (Vorechovsky et al., 1997; Evangelista et al., 2006) and to control the fate of tumor-initiating cells (Bar et al., 2007; Justilien et al., 2014; Takebe et al., 2015). Indeed, HH plays an essential role in driving tumorigenesis and drug resistance (Filbin et al., 2013), along with favoring cancer-initiating phenotype (Bar et al., 2007). HH can also enhance the expression of angiogenic factors, such as angiopoietins (Lee et al., 2007). Interestingly, we found that DHH does not seem to be involved in maintaining BTICs. Indeed, DHH silencing did not influence BTICs tumorspheres formation and viability. Conversely, DHH largely contribute to BTICs-CM impact on brain endothelial cell behavior *in vitro* and tumor angiogenesis *in vivo*. In keeping with this idea, it has been reported that HH nexus is instrumental in maintaining the blood–brain barrier integrity during both development and adulthood in mouse models (Alvarez et al., 2011). In this context, pharmacological inhibitors targeting Src kinase activity did not fully abrogate BTICs-CM-triggered endothelial behavior. Indeed, the partial blockade of endothelial permeability increase obtained by Src inhibition might be attributed to the presence of other pro-permeability factors in the BTICs-CM, among which are Semaphorin 3A and VEGF-A (Bian et al., 2007; Dwyer et al., 2012; Le Guelte et al., 2012; Treps et al., 2015). Meanwhile, hindering VEGF-R2 tyrosine kinase activity did alter BTICs-CM action on brain endothelial cells. Overall, these observations suggest that DHH might operate independently of VEGF-A in the tumor microenvironment. Thus, DHH might emerge as a novel pro-permeability factor and a novel candidate for anti-edema and anti-angiogenic therapeutic actions.

Whereas Ptch1 signaling is widely documented, Ptch2 *modus operandi* and functions continue to be elucidated. For instance, Ptch1 triggers both a canonical and non-canonical signaling pathways (Aberger et al., 2012), and can synergize with PI3K pathway to promote tumor growth and viability in GBM. Accordingly, blockade of both pathways simultaneously resulted in mitotic catastrophe and tumor apoptosis (Filbin et al., 2013). Here, we found that Ptch1 expression in endothelial cells was not affected by BTIC-CM challenge. Likewise, silencing Ptch1 fails to stop BTIC-mediated endothelial remodeling. Furthermore, SHH was not secreted by either BTICs or endothelial cells, suggesting that the canonical SHH/Ptch1 pathway does not function in the co-culture system. Interestingly, it has been recently documented that Ptch2 could suppress SHH signaling (Alfaro et al., 2014). In this scenario, Ptch2 expression in endothelial cells might counteract the effects of SHH locally secreted by other cell types from the tumor mass, such as astrocytes and stromal cells. Collectively, our data suggest that Ptch2 upregulation in endothelial cells and further ligand activation cause an imbalanced HH signaling, that can account for the observed vascular dysfunctions and barrier disruption, while sparing SHH positive impact on tumor cell expansion.

Our results demonstrate that targeting the DHH/Ptch2 nexus emerges as an appealing approach to curb vascular dysfunctions in GBM. Indeed, ablation of HH signaling may interfere with both angiogenesis and vascular leakage, and thus tumor growth. Nevertheless, further studies addressing the receptor upregulation mechanism and its elicited signaling route are required. Overall, our work unveils a role for DHH in heightened tumor angiogenesis and permeability, which may ultimately favor GBM progression, and thus places the DHH/Ptch2 nexus as a putative target for novel therapies directed against malignant stem-cell niches in GBM.

### Conflict of Interest Statement

The authors declare that the research was conducted in the absence of any commercial or financial relationships that could be construed as a potential conflict of interest.
